# Nd:YAG Laser for Effective Treatment of Subhyaloid and Sub–Internal Limiting Membrane Hemorrhages Secondary to Valsalva Retinopathy: A Case Series

**DOI:** 10.1155/crop/1890436

**Published:** 2026-10-30

**Authors:** Yong Zheng Wai, Kar Yong Chong, Elicia Tze Zhing Chieng, Nurhayati Abdul Kadir, Lik Thai Lim, Wai Seng Chiang

**Affiliations:** ^1^ Department of Ophthalmology Hospital Duchess of Kent Sandakan Sandakan Malaysia; ^2^ Faculty of Medicine University of Malaya Medical Centre Kuala Lumpur Malaysia um.edu.my; ^3^ Faculty of Medicine University of Malaysia Sarawak (UNIMAS) Kuching Malaysia unimas.my

## Abstract

**Background:**

Subhyaloid and sub–internal limiting membrane (sub‐ILM) hemorrhages secondary to Valsalva retinopathy typically occur in young adults and can cause sudden, significant visual impairment. Spontaneous resolution may be prolonged, and delayed clearance can adversely affect visual recovery. Nd:YAG laser membranotomy has been proposed as an effective, minimally invasive treatment to facilitate rapid visual improvement.

**Methods:**

This case series included three patients aged 24–27 years who presented with subhyaloid and sub‐ILM hemorrhages secondary to Valsalva retinopathy. The duration of symptoms prior to treatment ranged from 2 to 10 days. The mean horizontal and vertical diameters of the hemorrhages were 3615 and 3289 *μ*m, respectively. All patients underwent Nd:YAG laser membranotomy. The mean total laser energy applied was 79.7 mJ (range 11–185 mJ). Best‐corrected visual acuity (BCVA) and postoperative complications were assessed.

**Results:**

Preprocedure BCVA ranged from 6/9 to 1/60. Rapid anatomical and functional improvement was observed in all cases following treatment. At 2 weeks after the procedure, BCVA improved to 6/9 in all patients. No complications such as macular hole formation or retinal detachment were noted during the follow‐up period.

**Conclusion:**

Nd:YAG laser membranotomy appears to be a safe and highly effective treatment for subhyaloid and sub‐ILM hemorrhages secondary to Valsalva retinopathy, particularly in young patients requiring rapid visual recovery. Larger studies with longer follow‐up are needed to further evaluate long‐term outcomes and potential complications.

## 1. Introduction

Valsalva retinopathy, an infrequent condition, presents as an acute visual impairment caused by preretinal hemorrhages, usually near the macula, following a Valsalva maneuver. First reported by Thomas Duane in 1972, it is described as a hemorrhagic retinopathy resulting from a sudden increase in intrathoracic pressure [1]. The Valsalva maneuver involves a forced expiratory effort against a closed airway, leading to increased intrathoracic and intra‐abdominal pressure. The raised pressure reduces venous return and stroke volume, subsequently elevating systemic venous pressure. The resulting rise in intraocular venous pressure can cause the rupture of superficial retinal capillaries in the macula, leading to hemorrhages.

The possible anatomical locations for Valsalva retinopathy include subhyaloid, sub–internal limiting membrane (sub‐ILM), intraretinal, subretinal, and intravitreal [2]. Management strategies for this condition are diverse, encompassing observation, pneumatic displacement, green argon laser, and surgical intervention such as pars plana vitrectomy or neodymium‐doped yttrium aluminum garnet (Nd:YAG) laser treatment [3, 4].

Spontaneous resolution from observation alone can require an extended period, ranging from several weeks to months. Although pars plana vitrectomy offers rapid clearance, its invasive nature is a limiting factor. Conversely, Nd:YAG laser is recognized as an effective and less invasive alternative that facilitates quick visual improvement by clearing both subhyaloid and sub‐ILM hemorrhages [5, 6]. For young and productive patients, rapid visual recovery is crucial for their ability to contribute to their family and community. Nd:YAG laser is one of the most effective and noninvasive treatment options. In this case series, we discuss three cases of preretinal hemorrhages and their management with Nd:YAG laser in young adults without any underlying retinal diseases.

## 2. Case Presentation

### 2.1. Case 1

A 26‐year‐old lady, primigravida at 34 weeks and 6 days gestation, presented with sudden onset of left eye (OS) floaters for 2 days associated with inferior visual defect. She had reported a history of multiple episodes of vomiting prior to the onset.

Her visual acuity upon presentation was 6/9 in the OS. Anterior segment examination was unremarkable. Fundus examination revealed fresh subhyaloid and sub‐ILM hemorrhage over the superotemporal macula area involving the superotemporal arcade, sparing the fovea. (Figure 1A).

**Figure 1:**
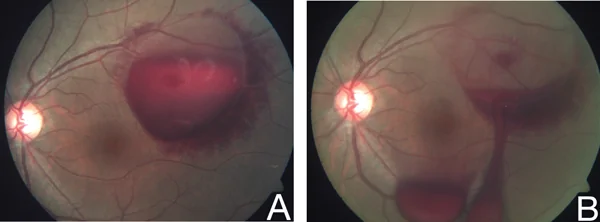
(A) Subhyaloid hemorrhage measured 3 DD (horizontal) × 2 DD (vertical) or 4050 × 3155 * μ*m. (B) Fundus photo taken immediately after Nd:YAG posterior hyaloidotomy shows rapid blood drainage to the vitreous cavity.

Although the hemorrhage spared the fovea—making conservative observation a viable alternative—the patient experienced severe anxiety regarding the dense inferior visual field defect. Being in her third trimester of pregnancy, she felt the scotoma was disorienting and significantly increased her risk of falls, particularly when navigating stairs. Following comprehensive counseling on the risks and benefits of both observation and laser intervention, the patient opted for Nd:YAG laser hyaloidotomy to achieve rapid visual rehabilitation and alleviate her safety concerns.

Nd:YAG (Visulas YAG III Combi, Carl Zeiss, Germany) posterior hyaloidotomy was performed on the same day with a Volk Area Centralis lens (Volk Optical, Inc., Mentor, Ohio), aimed over the inferior portion of the subhyaloid hemorrhage. Two shots with an energy of 5.5 mJ were given (total energy 11 mJ). Blood clot was seen draining into the vitreous (Figure 1B). After 1 week, visual acuity remained 6/9, with no inferior visual field defect.

### 2.2. Case 2

A 24‐year‐old lady presented with sudden‐onset OS blurring of vision after coughing 2 days ago. Her visual acuity upon presentation was 1/60. Fundus examination revealed sub‐ILM hemorrhage over the fovea involving the inferotemporal arcade (Figure 2A).

**Figure 2:**
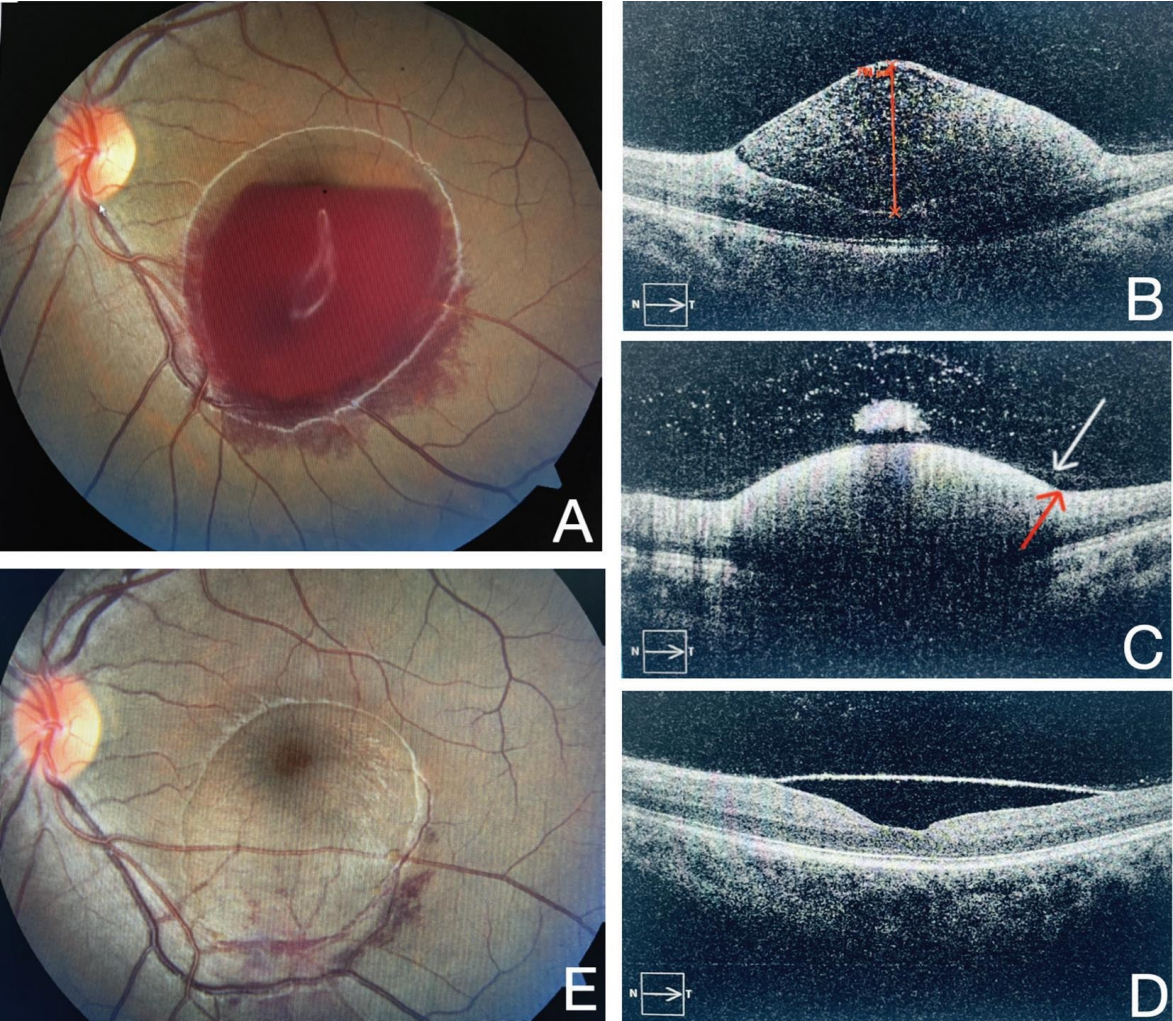
(A) Sub‐ILM hemorrhage measured 3 DD (horizontal) × 3 DD (vertical) or 3450 × 3525 * μ*m involving the fovea. (B) Sub‐ILM hemorrhage thickness of 791 *μ*m (red line) from the fovea, able to visualize the fovea contour vaguely below the blood. (C) OCT taken immediately after membranotomy shows sub‐ILM blood escaped from the puncture site into the vitreous; the white arrow shows the posterior hyaloid, and the red arrow shows the ILM layer. This highlights the location of hemorrhage at the sub‐ILM layer rather than subhyaloid. (D) OCT of the fovea 1 week after Nd:YAG membranotomy revealed empty sub‐ILM space with similar fovea contour compared with before membranotomy. (E) Fundus photo 1 week after membranotomy shows crescent‐shaped border of the ILM.

Preprocedure optical coherence tomography (OCT) of the macula was performed to measure the prefoveal blood thickness to ensure sufficient thickness of blood to cushion the impact of the Nd:YAG laser (Figure 2B).

She underwent Nd:YAG laser (Visulas YAG III, Carl Zeiss, Germany) membranotomy aimed at the inferior portion of the sub‐ILM hemorrhage on the same day. Initial attempts using the Volk Area Centralis lens were unsuccessful in penetrating the ILM despite eight consecutive shots with power ranging from 5.5 to 12 mJ. The procedure was then transitioned to a Volk SuperField lens (Volk Optical, Inc., Mentor, Ohio). This switch provided a different laser spot magnification profile and focal point, allowing successful penetration of the ILM after seven shots using the same energy range (5.5–12 mJ). A total cumulative energy of 185 mJ was delivered across 15 shots (eight initial unsuccessful shots using the Volk Area Centralis lens, followed by seven successful shots after switching to the Volk SuperField lens). The maximum single‐shot energy used was kept within established safe thresholds (12 mJ per pulse). Maximal energy per shot delivered was 12 mJ. OCT over the point of membranotomy was taken immediately. The OCT also helps to localize the blood and to differentiate subhyaloid and sub‐ILM hemorrhage accurately, where the white arrow shows the posterior hyaloid and the red arrow shows the ILM layer (Figure 2C). OCT macula and fundus photos were repeated after 1 week (Figure 2D,E). Her uncorrected visual acuity improved to 6/9 after 1 week.

### 2.3. Case 3

A 27‐year‐old lady presented with sudden blurring of vision in her OS for the past 10 days after she laughed. The blurring of vision was abrupt and occurred immediately after her laughter. Her visual acuity upon presentation was 1/60. Fundus examination revealed sub‐ILM hemorrhage over the central macula. Fundus autofluorescence (FAF) was taken before the procedure (Figure 3A,B).

**Figure 3:**
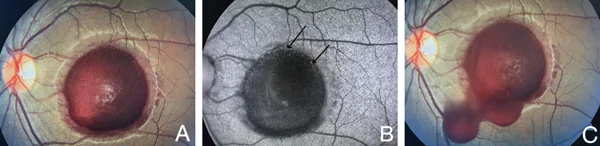
(A) Sub‐ILM hemorrhages measure 2 × 2 DD or 3345 × 3187 * μ*m located at the central macula. (B) FAF shows hypoautofluorescent hemorrhages with speckles of hyperautofluorescent spots (black arrows) at the border of sub‐ILM hemorrhages, suggestive of more chronic, dehemoglobinized blood. (C) Sub‐ILM blood drained into the vitreous cavity immediately after Nd:YAG membranotomy.

Nd:YAG laser (Visulas YAG III, Carl Zeiss, Germany) membranotomy was performed on the same day with the Volk Area Centralis lens (Volk Optical, Inc., Mentor, Ohio), aimed at the inferior portion of the sub‐ILM hemorrhage. A total of seven shots were delivered with energy ranging from 5.5 to 8 mJ per shot, for a total energy of 43.1 mJ. A fundus photo was taken immediately after Nd:YAG membranotomy (Figure 3C). Her uncorrected visual acuity improved to 6/9 1 week after Nd:YAG membranotomy.

## 3. Discussions

Valsalva retinopathy is an infrequent condition that occurs due to a sudden increase in intrathoracic pressure. The etiology of Valsalva retinopathy involves a sudden surge in intrathoracic pressure, typically caused by lifting, vomiting, straining, or coughing [2]. We report a case series that includes a rare instance of Valsalva retinopathy triggered by laughter.

The sudden blurring of vision is due to premacular hemorrhage. The blood is commonly trapped in the subhyaloid, sub‐ILM, or both [7]. Clinically, a “double‐ring sign” can be seen if the blood is trapped in both spaces. The outer ring represents subhyaloid hemorrhage, and the inner ring represents sub‐ILM hemorrhage [8]. In cases of doubt, OCT can be a very useful tool to identify the location of the hemorrhage. The more reflective layer is determined as the ILM, and a layer of low optical reflectivity above the ILM is the posterior hyaloid, as shown in Figure 2C [7]. Although OCT is the gold standard for precisely localizing preretinal hemorrhages, it was unfortunately unavailable for Case 1 and Case 3 due to unexpected equipment malfunction at the time of presentation. Consequently, the anatomical location of the hemorrhages in these cases was determined based on classic clinical features, such as the double‐ring sign, and detailed biomicroscopic fundus examination.

OCT can accurately measure the hemorrhage volume and size; however, no literature investigates the safe thickness of hemorrhage to proceed with Nd:YAG membranotomy. One of our cases had a blood thickness of 791 *μ*m from the fovea (Figure 2B), and no retinal scarring was noted after membranotomy. An adequate cushion of blood thickness can play a protective role for the underlying neurosensory retina. Further study is required to determine the minimal thickness of hemorrhage needed to proceed with Nd:YAG membranotomy.

Current management protocols for Valsalva retinopathy include conservative observation, Nd:YAG laser treatment, and pars plana vitrectomy. Observation takes around 99 days for the hemorrhage to resolve [2]. Smaller hemorrhages resolve faster compared with larger ones; hemorrhages of 1 disc diameter (DD) or smaller can resolve within 2–3 months [9]. Some studies advocate observing for up to 3 months for spontaneous resolution, whereas others incline toward early intervention to prevent irreversible retinal damage caused by hemosiderin [10].

Nd:YAG laser was manufactured for anterior segment procedures such as capsulotomy and iridotomy. Faulborn was the first to use Nd:YAG posterior hyaloidotomy for premacular hemorrhage [11]. The term “laser posterior hyaloidotomy” is used for subhyaloid hemorrhage and “laser membranotomy” for sub‐ILM hemorrhage. Laser posterior hyaloidotomy or membranotomy is a simple, effective, office‐based procedure to recover vision in patients with premacular hemorrhage. Valsalva retinopathy is commonly seen in the working‐age group; rapid vision recovery enables them to return to the workforce earlier.

Controversy remains regarding the ideal timing for laser membranotomy. Some authors recommend performing Nd:YAG membranotomy within 3 weeks after the onset of hemorrhage [10, 12, 13]. The effectiveness of YAG membranotomy is uncertain for older hemorrhages where blood clotting or dehemoglobinization is present. Fresh hemorrhage typically appears hypoautofluorescent due to the absorption of excitation light by intact red blood cells. A large blood clot might not be able to drain through the small laser membranotomy site [14]. Hence, FAF is a useful tool to detect signs of dehemoglobinization. Over time, hemoglobin degrades into bilirubin, an endogenous fluorophore with an excitation peak near 470 nm and emission around 520 nm, which manifests as hyperautofluorescent speckles on FAF. The presence of these hyperautofluorescent signals indicates significant dehemoglobinization and blood clotting. This indicates the chronicity of the hemorrhage, as shown in Figure 3B. Khadka et al. successfully performed laser membranotomy in two patients whose symptoms had persisted for over 45 days [6]. All three patients in our case series, who presented within the crucial 3‐week window, demonstrated a strong, complication‐free response to Nd:YAG laser hyaloidotomy or membranotomy.

Reported energy levels for Nd:YAG laser membranotomy vary widely in the literature, ranging from 1 to 50 mJ per shot [6, 9, 15]. Consequently, the optimum energy setting remains debated, largely due to variations in hemorrhage density and chronicity. Ulbig et al. recommended a safe limit of 9 mJ [16]. Gabel et al. observed no retinal damage even at energy levels up to 50 mJ per shot [15]. Advocating for a more conservative approach, Khadka et al. suggested an initial energy setting of 5 mJ, with gradual 1 mJ increments up to a maximum of 12 mJ [6]. More recently, successful outcomes have been reported using even lower energy settings; Hernández‐Emanuelli et al. achieved rapid visual recovery using 1.8 mJ bursts for a subhyaloid hemorrhage, and Larrea et al. managed a sub‐ILM hemorrhage with just two shots of 2.5 mJ after precise OCT localization [17, 18].

A critical distinction must be made between single‐shot peak energy and cumulative total energy when assessing procedural safety. Photodisruptive retinal injury is predominantly dictated by single‐pulse focal energy (mJ) and localized power density, rather than the total cumulative energy delivered across multiple pulses. In Case 2, although a high cumulative energy of 185 mJ was required across 15 total shots due to initial lens incompatibility, the single‐shot energy was strictly capped at a maximum of 12 mJ per pulse. The primary safety buffer protecting the underlying outer retina and RPE from photodisruptive shockwaves was the substantial prefoveal blood cushion (791 *μ*m on OCT). This dense hematoma effectively absorbed and attenuated acoustic energy waves. Switching from the Volk Area Centralis to the Volk SuperField lens altered the focal spot magnification profile and energy density at the target membrane, successfully achieving ILM disruption without exceeding safe single‐shot power limits.

Although Nd:YAG membranotomy is minimally invasive, potential complications include epiretinal membrane (ERM) formation, macular tractional detachment, and a persistent premacular cavity [6, 9]. Furthermore, selecting an inappropriately high energy setting increases the risk of retinal hole formation, though Suren et al. reported a case of a laser‐induced macular hole that closed spontaneously 6 months after the procedure [19]. To minimize these risks, ensuring an adequate cushion of blood is essential to provide a protective barrier for the underlying retina. Consistent with established safety parameters, all procedures in our series were executed successfully using a per‐shot energy range of 5.5–12 mJ, reaching a maximum total energy of 185 mJ, and were entirely free of complications.

Although Nd:YAG membranotomy provides rapid central visual decompression, long‐term structural and vitreous consequences must be considered. Mechanical detachment of the ILM or posterior hyaloid by a large premacular hematoma can leave a persistent anatomical “retinal footprint” [3, 7]. Long‐term OCT studies have demonstrated microstructural sequelae, including persistent premacular cavities, ILM rugae, hyperreflective membrane folds, and localized RPE alteration secondary to hemosiderin toxicity [3, 14, 18]. Furthermore, long‐standing ILM separation can act as a scaffold for glial cell proliferation, predisposing eyes to secondary ERM formation [14, 18].

Additionally, draining premacular blood effectively converts a localized, encapsulated preretinal collection into a diffuse vitreous hemorrhage [5, 16]. In young, phakic individuals with a firm vitreous gel and an attached posterior hyaloid, clearance of this gravity‐dependent vitreous blood can be prolonged over weeks to months, resulting in persistent symptomatic floaters and transient visual degradation until complete reabsorption occurs [6, 13, 14, 16].

Overall, our patient demographics and clinical presentations closely mirror contemporary reports, affirming that Valsalva retinopathy primarily affects healthy, young individuals following sudden physical exertion. Similar to recent findings, we also found OCT to be invaluable for precisely localizing the hemorrhage to the sub‐ILM space, as demonstrated in Case 2. Although our cases necessitated slightly higher energy parameters (5.5–12 mJ) than the 1.8–2.5 mJ bursts highlighted in recent low‐energy reports, this variability suggests that the required laser energy heavily depends on specific membrane thickness, hemorrhage chronicity, and the exact focal point of the lens used. Ultimately, despite utilizing slightly higher energy in our series, the protective blood cushion ensured complete drainage without retinal damage, which aligns perfectly with the excellent safety profiles documented in the current literature.

There are few universal recommendations for performing a posterior Nd:YAG laser membranotomy. It is advisable to select a drainage site that is sufficiently distant from the inferior border where there is significant hemorrhagic elevation. Additionally, it is sensible to choose a drainage area away from the fovea and major blood vessels. Depending on the location of the hemorrhage and to avoid the fovea, it may sometimes be safer to drain blood from the lateral aspect of the hemorrhage. Positioning the patient′s head so that the blood will drain into the vitreous cavity under the influence of gravity can also be beneficial. This case series shows Nd:YAG laser has good outcomes in subhyaloid and sub‐ILM hemorrhages secondary to Valsalva retinopathy among young patients.

With the evolution of 25‐ and 27‐gauge microincision vitrectomy surgery (MIVS), primary surgical intervention has gained significant traction [2, 4, 10]. Modern MIVS represents a definitive, one‐step surgical solution that guarantees immediate and complete hematoma evacuation, eliminates slow‐resolving vitreous hemorrhage, and allows simultaneous ILM peeling to prevent posthemorrhagic ERM formation or macular traction [4, 9, 10].

However, whether MIVS should supersede Nd:YAG laser as the primary management option depends on patient‐specific factors. Nd:YAG membranotomy remains a compelling, minimally invasive, cost‐effective, and office‐based first‐line alternative for fresh (< 3 weeks), nonclotted premacular hemorrhages with an adequate protective blood cushion [6, 9, 14]. It avoids operating room dependencies, surgical morbidity, and the rare but severe risks of intraocular surgery (e.g., endophthalmitis, iatrogenic retinal tears, and premature cataractogenesis in young phakic patients) [2, 6, 14]. Therefore, we propose a tailored approach: Nd:YAG laser membranotomy is ideal as a primary, noninvasive first‐line intervention in acute, nonclotted cases, whereas 25G/27G MIVS should be considered the primary treatment for chronic/clotted hemorrhages, cases with thin protective blood cushions, or as a definitive secondary procedure when laser drainage fails [2, 4, 9].

Proper case selection is paramount for optimizing clinical outcomes in Valsalva retinopathy, given the diverse management options available. Conservative observation is most appropriate for small hemorrhages of 1 DD or smaller, which typically undergo spontaneous resolution within 2–3 months [9]. Conversely, Nd:YAG laser membranotomy is the preferred first‐line intervention for fresh, nonclotted hemorrhages presenting within the crucial 3‐week window after symptom onset. It is particularly indicated for young, working‐age patients who require rapid visual rehabilitation to return to their daily routines, provided the hemorrhage is large or thick enough to offer a protective cushion for the underlying neurosensory retina. Pars plana vitrectomy, on the other hand, should be reserved for chronic cases where significant dehemoglobinization and blood clotting prevent effective laser drainage or when intervention is delayed beyond the optimal timeframe.

The main limitation of this case series is the very small sample size (*n* = 3), which limits the generalizability of the findings. In addition, the absence of a control or comparison group restricts our ability to definitively assess the true efficacy of the Nd:YAG intervention compared with natural progression. Furthermore, this case series lacks long‐term follow‐up data; therefore, late complications such as ERM formation and macular hole development may have been missed. Consequently, larger, well‐designed studies with adequate follow‐up are warranted to more accurately evaluate the outcomes of Nd:YAG laser membranotomy in Valsalva retinopathy.

## 4. Conclusions

As observed in our case series, patients with Valsalva retinopathy characterized by hemorrhages of 2 DD or larger and presenting within 3 weeks of symptom onset are good candidates for Nd:YAG laser membranotomy. Using an energy level between 5.5 and 12 mJ per shot is considered safe. It is essential to aim the laser at the inferior area where there is sufficient blood thickness to cushion the photodisruption, thereby minimizing the risk of complications. In conclusion, Nd:YAG laser membranotomy is a cost‐effective, safe, and efficient treatment option for subhyaloid or sub‐ILM hemorrhage secondary to Valsalva retinopathy in young adults.

## Author Contributions

Y.Z.W., K.Y.C., and E.T.Z.C. gathered the necessary information about the patient. Y.Z.W. wrote the manuscript under the supervision of N.A.K., L.T.L., and W.S.C. L.T.L. did a thorough revision of the work. N.A.K. and W.S.C. are the main consultants who managed the patients.

## Funding

No funding was received for this manuscript.

## Disclosure

All authors have read and approved the final version of the manuscript. All authors agreed to submit the manuscript to *Case Reports in Ophthalmological Medicine*. Y.Z.W. affirms that this manuscript is an honest, accurate, and transparent account of the study being reported, that no important aspects of the study have been omitted, and that any discrepancies from the study have been explained.

## Ethics Statement

This case series is registered under the Malaysian National Medical Research Registry (NMRR ID‐24‐02489‐8SN), which follows the tenets of the Declaration of Helsinki.

## Consent

Written informed consent was obtained from the patients for participation and for publication of this case series and any accompanying images.

## Conflicts of Interest

The authors declare no conflicts of interest.

## Data Availability

All data and materials gathered during this study are included in this study. Data sharing is not applicable to this article as no datasets were generated or analyzed during the current study.
